# A Cognitive Behavioral Model Proposing That Clinical Burnout May Maintain Itself

**DOI:** 10.3390/ijerph18073446

**Published:** 2021-03-26

**Authors:** Niclas Almén

**Affiliations:** Department of Psychology and Social Work, Mid Sweden University, 831 25 Östersund, Sweden; niclas.almen@miun.se

**Keywords:** prolonged stress, recovery, burnout, exhaustion, maintenance

## Abstract

Burnout is common in many countries and is associated with several other problems such as depression, anxiety, insomnia, and memory deficits, and prospectively it predicts long-term sick-leave, cardiovascular disease, and death. Clinical burnout or its residual symptoms often last several years and a common assumption is that recovery takes a long time by nature, despite full time sick-leave and the absence of work stress. The literature suggests models that hypothetically explain the development, but not maintenance, of the syndrome. Based on cognitive and behavioral principles, stress research, and stress theories, this paper describes a theoretical model explaining how clinical burnout can develop and be maintained. While the development of clinical burnout is mainly explained by prolonged stress reactions and disturbed recovery processes due to work related stressors, maintenance of the syndrome is particularly explained by prolonged stress reactions and disturbed recovery processes due to the new context of experiencing burnout and being on sick-leave. Worry about acquired memory deficits, passivity and excessive sleep, shame, fear of stress reactions, and the perception of not being safe are examples of responses that can contribute to the maintenance. The model has important implications for research and how to intervene in clinical burnout. For example, it can offer support to professional care providers and patients in terms of focusing on, identifying, and changing current contextual factors and behaviors that maintain the individual’s clinical burnout symptoms and by that facilitate burnout recovery. Regarding research, the model provides a highly important reason for researchers to study contextual factors and behaviors that contribute to the maintenance of clinical burnout, which has been neglected in research.

## 1. Introduction

Toward the end of the millennium, long-term sick-leave due to severe burnout escalated dramatically in Sweden. At that time, I was responsible for creating a rehabilitation program for people suffering from severe, usually prolonged, burnout symptoms and being on sick-leave due to these symptoms. When I consulted the literature on burnout, it became clear to me that it would have been very helpful if we had been working with prevention and not rehabilitation, since it focused on the risk factors for developing, but not maintaining, the syndrome. In line with this, most clients at the clinic focused on historical stress variables that could not be changed. As a consequence, I developed a theoretical model that hypothetically not only explained the development but also the maintenance of severe/clinical burnout. The aim of the model was to show that every participant probably had current factors in their lives that influenced their burnout levels and that could be changed. The theoretical model worked as a therapeutic intervention, for example, it gave many people a sense of control and hope, and guidance on what to do in the present, rather than information on what they should have done in the past. The model also motivated participants who were passively waiting for better health to be more active and health goal-oriented. The model has been used in clinical practice, university courses, and in intervention research [1]. However, the model has only been available to Swedish speaking persons, since it has only been published in a Swedish article [2] and in a Swedish book [3,4].

Although there exists different definitions and measures of burnout, the common feature among them is *exhaustion*. Pines, Aronson, and Kafry [5] have been influential in their definition of burnout as non-transient physical, emotional, and mental exhaustion. In line with this, burnout has been considered a chronic depletion of an individual’s energy resources [6] manifested by prolonged feelings of physical fatigue, emotional exhaustion, and cognitive weariness [7]. The concept of chronic or non-transient exhaustion/energy depletion should not be taken literally. People experiencing burnout are usually not *always* exhausted. Rather, they experience a reduced capacity to tolerate stress/demands, lack of endurance, exhaustibility, and increased time needed for recovery after stress and effort [8]. A marked inability to cope with demands at work and outside work due to severe levels of these symptoms is the definition of clinical burnout in this paper. Some definitions of burnout also include other factors. One of the most used definitions of burnout formulated by Maslach and Jackson [9] includes three aspects: emotional exhaustion, and two possible consequences of this: cynicism and a reduced ability to perform at work. Another definition of burnout includes withdrawal behavior as part of the syndrome [10]. When measuring burnout, many researchers include tension and listlessness (i.e., low levels of interest in doing things) [11,12,13], although the founders of the subscales measuring tension and listlessness described these as burnout concomitants, and not burnout per se [14]. 

Burnout is problematic in many countries worldwide. People with burnout usually have multidimensional symptoms/problems, in particular anxiety and depression [15]. Approximately half of those defined as severely burned-out have been shown to be clinically depressed [16]. Burnout is also associated with somatic discomfort and dysfunctions (e.g., digestive problem, skin problems, and headaches) [17], reduced reproductive functions, type 2 diabetes, cardiovascular disease, and cardiovascular-related events [18]. Although burnout symptoms can be reduced over time, they often persist for several years [19], particularly among people who have been diagnosed as clinically burned out, and the symptoms are associated with long-term illness [20] and predict all-cause mortality [21]. A study by Glise et al. [19] showed at a seven-year follow-up that a third of former patients with exhaustion disorder (i.e., clinical burnout) were clinically exhausted and only 16% reported that they were fully recovered. Moreover, patients who consider themselves to be fully recovered may still have symptoms such as memory problems or sleep problems. Burnout predicts permanent work disability [22], and long-term sickness absence due to clinical burnout, which may be an indicator for premature mortality [20]. A factor that usually has significant negative consequences for a person scoring high levels of burnout, not the least in terms of work ability, is cognitive impairment and in particular in terms of attention, memory, and executive functions [23]. Apart from significant consequences for the individual, work-related stress is enormously costly for our society [24]. 

Many people recover poorly from burnout despite full time sick-leave for long periods of time. One common assumption is that clinical burnout symptoms “by nature” require long recovery time, perhaps several years [25]. It has been suggested that the chronicity of clinical burnout syndrome may possibly be explained by chronic changes in biological functions including brain functions [19]. However, there are no biomarkers for clinical burnout [26], and thus no evidence for such an explanation or for clinical burnout lasting for many years, despite the absence of current significant stressors and stress behaviors. Accordingly, I hypothesize that clinical burnout is in many cases partially or fully maintained by current contextual and behavioral factors. 

Research has to a very large degree focused on the association between stress and burnout, and researchers have highlighted the importance of further increasing the knowledge on the progression of burnout and its pathways to sick-leave [27]. However, maintenance of burnout has been neglected in research. 

Cognitive behavioral therapy (CBT) is the current gold standard of psychotherapy [28]. A characteristic aspect of CBT is the use of a theoretical model explaining not only the development but also, and primarily, the maintenance of clinical disorders. Generally, these are based on learning theory/behavior analysis and/or cognitive information processing theory, and theories and empirical data on the analyzed disorder. According to many CBT models, temporary reactions (such as anxiety or low mood) are unproblematic consequences of natural events in life, while chronicity are consequences of maladaptive coping. For example, a CBT model for depression states that depression can develop as a consequence of losses in life (e.g., to lose a close relative or a job), while long-term depression is mainly explained by behavioral patterns of inactivity, withdrawal, and avoidance [29,30]. Another problem is long-term pain, which according to a CBT model is explained by the individual’s fear of pain, and pain avoiding behaviors, which may hinder recovery from the acute pain, and create new problems (e.g., physical disuse, depression, and disability) [31]. A further example is insomnia: Temporary sleep problems can be caused by loud neighbors or transient stress at work, while prolongation of sleep problems may occur as a consequence of worry about sleep and conditioned arousal in the sleep situation [32]. A last example is panic attacks. The first panic reaction an individual experiences may be due to a period in life that is very stressful, whereas repeated panic attacks may be caused by fearful thoughts regarding normal physical reactions (e.g., due to walking up the stairs) [33]. Cognitive behavioral therapy emphasizes the intervention of maintaining factors.

The aim of the present paper is to, on the basis of a CBT-approach (i.e., learning theory/behavioral analysis, cognitive information processing theory, and theory and empirical data regarding the analyzed problems) describe a theoretical model that aims to hypothetically explain the development and maintenance of clinical burnout. The primary purpose of the model is as follows: To address and emphasize clinical burnout maintaining factors and thereby facilitate recovery from the syndrome. 

## 2. A Proposed Cognitive Behavioral Explanatory Model of Clinical Burnout

There are many factors that constitute a risk as well as protective factors for developing and maintaining burnout. The proposed theoretical framework presented below is based on the assumption that burnout (i.e., exhaustion) syndrome is a consequence of prolonged and/or high frequent stress physiological responses [34] and insufficient recovery responses (during the day [35] and during sleep [36]). Furthermore, the assumption is that these responses are related to contextual factors and cognitive and overt behaviors. The model focuses primarily on (overt and covert) behaviors since these usually constitute the primary target in evidence-based treatments [37]. The stress-vulnerability hypothesis is acknowledged [38]: The levels of stress and lack of recovery that are required for burnout to develop and be maintained differs among people. The model suggests that factors that contribute to the development of clinical burnout may be different than those that contribute to the maintenance of the syndrome. Although the model is primarily a framework, specific factors within the framework will be suggested as important to the risk of developing and/or maintaining the syndrome. 

### 2.1. Stressors and Resources

A common feature of many established definitions of a stressful situation is an imbalance between stressors/demands and resources [39]. There is a large number of factors that have been shown to function as stressors. In particular, work-related factors such as work content, work characteristics, work organization, and social relations have been studied in relation to, and have been shown to covary with stress and health/well-being. For example, a systematic review including meta-analysis of work environment and burnout symptoms by Aronsson et al. [40] showed that high levels of different types of demands, a high workload, and job insecurity increased the risk of developing exhaustion. Further examples of stressors at work that may contribute to burnout are sex discrimination and role conflicts [41]. Both work stressors and nonwork stressors are important to burnout [41], although the former seems to have a greater impact [42]. In a study by Hasselberg [42], quantitative demands at work were by far the most common self-reported stressor in a sample of people with stress-related exhaustion. The second most common self-reported stressor was relationship conflicts in a person’s private life, and the third most common self-reported stressor was emotional demands at work. Additionally, work–family conflicts are important for stress reactions [43] and burnout [44]. An increasing number of individuals struggle with the challenges of fulfilling responsibilities at home and at work [45], which can lead to work-family conflict (i.e., stress reactions both during the day and evening with few opportunities for stress recovery). 

A common factor between the (potential) stressors described above is that they put demands on the person. In general, a critical aspect of these factors is not primarily that they may trigger stress responses per se, but prolonged or high frequent responses, which prevents sufficient recovery between these responses [34]. Some stressors such as anger provoking stressors are more associated than others with post-stressor sustained stress activation [46]. 

A fundamental resource for handling stressors is energy to use appropriate coping behaviors. Apart from that, the study by Aronsson et al. [40] showed that high levels of job support, workplace justice, job control, and reward helped protect against exhaustion. Further examples of stressor/demand relevant resources are self-efficacy [47], having a transformative leader at work [48], and formal education relevant for the work and position at work [41]. 

**Figure 1:**
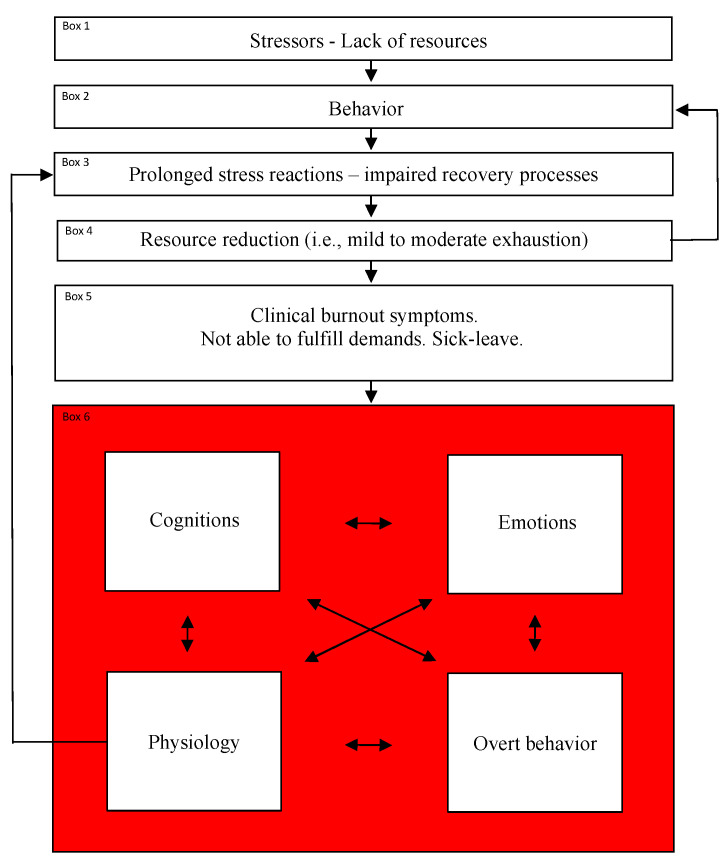
A proposed cognitive behavioral explanatory model of clinical burnout.

Stressors and resources are represented by box 1 included in the visualization of the model (see Figure 1). Pre-burnout behaviors, which will be addressed below, are represented by box 2.

### 2.2. Behaviors

A critical factor in explaining whether situations characterized by an imbalance between stressors/demands and resources lead to burnout is how the individual behaves before, during, and after these situations. The most fundamental aspects of the behavior concern *stress reactivity* and *stress recovery* [49]. As already stated, the present model hypothesizes that clinical burnout is not a consequence of physiological stress responses per se, but prolonged and/or a high frequency of such responses [34] and insufficient recovery responses (see box 3 in Figure 1).

According to the Selyean view, the stress response is defined as the increase in unspecific physical activation (i.e., arousal) that follows as a response to any type of demand/stressor [50]. According to Lazarus and Folkman [51], the stress response is dependent on the perception of danger, whereas the recent theory, the *generalized unsafety theory of stress*, states that lack of perception of safety is enough for stress reactions to occur [52], which is related to *potential* danger. An essential physiological aspect of a stress response is the activation of the sympathetic nervous system [53] and the altered hypothalamus–pituitary–adrenal (HPA) axis regulation [54]. Initially, the stress response referred only to automatic physiological responses, while the term has also begun to be used for other stress relevant responses, for example “voluntary” (i.e., operant) coping behavior. 

Stress recovery is a process of psychophysiological deactivation after effort expenditure [55]. If conceptualizing stress recovery as the opposite to stress according to the Selyean view [50], it can be described as the responses decreasing in unspecific physical activation (i.e., arousal), which follows as a response to the removal of any type of demand. Additionally, increased levels of parasympathetic vagal activity is associated with stress recovery [56]. A significant recovery outcome is the restoration of energy and ability to deal with future demands [57]. Recovery includes both automatic physiological responses and “voluntary” (i.e., operant) coping behavior [35,58].

It is important to point out that even if the duration of the stress situation is low, the stress responses can be prolonged due to cognitive behaviors such as worry and rumination for a long period of time before as well as after the external stress situation [59]. How people behave/cope in relation to their environment such as stressors varies [60]. This variability can be explained by conditioning (i.e., learning) and cognitive information processing. Although a unidirectional perspective on behavior and external factors (external factors as antecedents for behavior) was the focus in the present paper, these factors interact. 

Folkman and Lazarus [61] define coping as cognitive and behavioral efforts to manage (i.e., master, reduce, or tolerate) a troubled person–environment relationship. Adaptive coping can be viewed as having two major functions: the regulation of distressing emotions (so called emotion-focused coping) and the altering of whatever is causing the distress (so called problem-focused coping). Notably, this view, which was described by Folkman and Lazarus 1980 [62] has been very well-used since then. However, it does not incorporate recovery facilitating behavior, which has recently been demonstrated to be a critical behavior in an individual’s ability to cope with stress and maintain health and well-being [35] as well as for depleted resources to recover [63]. Another, and perhaps more important distinction, is between approach coping and avoidant coping [60]. In a clinical situation, it is useful to distinguish between adaptive and maladaptive coping. However, what type of coping is adaptive? This depends on the context [64]. However, some coping behaviors are generally adaptive whereas others are generally maladaptive. Approach coping in particular (such as problem solving) is positively related to health and well-being, while avoidant coping is negatively related to health and well-being [65]. Accordingly, CBT usually consists of reducing avoidance coping behaviors and increasing approach coping behaviors, for example, using exposure [66] and behavioral activation techniques [30].

There are some general patterns of behavior that may be seen as maladaptive coping behaviors since they are associated with stress reactions, recovery deficiencies, and ill health. An example of such behavior is *overcommitment to work* [67]. This behavior includes a high need for control and approval, continuous work commitment, high achievements, and increased levels of stress reactions, for example, indicated by higher levels of norepinephrine and cortisol [68]. Overcommitted individuals are prone to overwork and exhaust their own resources [54]. A critical behavioral component of the behavior pattern for developing exhaustion may be low levels of detachment from work [54,69]. 

A behavior related to overcommitment to work is *type A behavior*, which can be described as including high frequencies of the following behaviors: (1) performance and competitive behaviors; (2) perceived time pressure and being in a hurry; (3) hostility, anger, irritability, and impatience [4]. Type A behavior predicts burnout [70]. The critical question is whether the behavior involves prolonged and/or high frequent stress physiological reactions and impaired recovery processes or not. The most toxic component of type A behavior may be factor three [70]. Both inhibited and expressed anger is associated with slow cardiovascular stress recovery and persistently low parasympathetic activation [71]. 

A further example of behavior that is hypothetically a risk factor for developing as well as maintaining burnout is *perfectionism*. This behavior is defined by high internal demands in many situations in life and a lack of flexibility in terms of adapting internal demands to different situations. People with such behaviors tend to ruminate on failures [72], which may jeopardize recovery in post-stress situations [59]. Correlations have been found between perfectionist tendencies and perceived stress, burnout, and psychopathological symptoms [73]. Chang [74] differentiates between adaptive and maladaptive perfectionism. The former includes a strong desire to perform at a high level, perseverance, and experience of satisfaction and desire when things are going well, while the latter is characterized by unrealistic goals and a fear of failure and criticism. In conclusion, excessive, and in particular fear-related, effort seems to be a risk factor for developing burnout. Accordingly, excessive efforts aimed at reducing fear of losing a relationship predicts burnout [75].

Stressors often lead to *perseverative cognitions*, most often measured in terms of prolonged worry and rumination [59]. The perseverative cognition hypothesis suggests that perseverative cognition can prolong physiological (stress) activation and thereby moderate the health consequences of stressors. Perseverative cognitions in post achievement-related stress situations are critical for the deterioration of well-being/health, particularly among people who are excessively achievement-oriented and highly self-critical [76]. Perseverative cognition indicates repetitive or sustained activation of cognitive representations of past stressful events or feared events in the future [77]. In daily life, most prolonged physiological activity is not due to stressful events but to perseverative cognition about them. Since we are basically capable of worrying and ruminating about anything at any time, and since worry is controlled by the principle of “better safe than sorry” [78], the total time of worry/rumination and accompanied physiological stress responses can be highly frequent and long lasting and be an obstacle to recovery [79]. Worry/rumination can also interfere with sleep and sleep quality during subsequent nocturnal sleep [80], which is particularly problematic since sleep is important for recovery from daily strain, to prevent exhaustion [81] as well as to recovery from burnout [82]. Worry/rumination can also influence overt “voluntary” recovery facilitating behaviors. For example, during a work break, a coworker who is worried about a future task may use the break for task preparation instead of doing something more relaxing. Of further importance for health in general, a systematic review and meta-analysis demonstrated that increases in perseverative cognitions in terms of rumination are associated with increases in health risk behaviors (i.e., substance use, alcohol consumption, unhealthy eating, and smoking) [83].

Recovery facilitating behaviors are important both at work and outside work [63] For example, a randomized control trial investigating an intervention aiming at strengthening recovery behaviors at work and outside work, without addressing or intervening stressors or stress responses, demonstrated large effect sizes on perceived stress, tension, anxiety, depression, and burnout [35]. Accordingly, a study by Söderström et al. [36] demonstrated that sleeping less than six hours per night and preoccupation with thoughts of work during leisure time, a factor disturbing stress recovery processes [84], prospectively predicts burnout [36]. In line with this, recovery from burnout has been shown to be accompanied by improved sleep continuity [82]. There are many contextual and behavioral factors that have a positive effect on stress recovery responses such as using a tension releasing technique [13], focused attention [85], physical exercise and aerobic fitness [86,87,88], sport activities [89], low levels of self-regulation [90], cultural activities [91], natural environments [92], listening to classical music [93], psychological detachment from work, relaxing activities, opportunities to decide what to do, and challenging activities [94,95]. Paradoxically, when we need recovery facilitating behaviors the most, they can be the most difficult to achieve [96]. 

### 2.3. A Vicious Circle

According to the current proposed theoretical model, the burnout process may be triggered by non-transient increasing demands (for example additional tasks at work) and (or) non-transient decreasing resources (for example reduced number of colleagues at work), and coping through increased effort to be able to handle the changed situation (for example, working during lunch breaks and work-related thoughts after the work day is over). This likely leads to increased stress reactions (i.e., arousal), and/or decreased recovery processes followed by suboptimal resource recovery. The latter, manifested by fatigue, results in reduced (internal) resources, which may lead to further increased effort. Thus, a vicious circle may develop and subsequent exhaustion may follow. The vicious circle may continue as long as the individual has the capacity to gradually increase their effort to deal with current demands. This is possible to do if the level of exhaustion is mild to moderate (represented by box 4 in Figure 1). Subsequently, clinical levels of exhaustion (represented by box 5 in Figure 1) are at risk of occurring, which is defined as a psychological and/or physiological inability to further mobilize effort in order to handle the demands that the person perceives in life. 

The behavioral patterns described above (overcommitment to work, type A behavior, and maladaptive perfectionism) are assumed to increase the probability for overuse of increased effort as a way to cope with a nonbeneficial change regarding stressors/demands and/or resources. According to a cognitive therapy approach [97], it is plausible that beliefs about oneself stating that all demands in life must be met, for example, in order not to interpret oneself as a defective person and experience accompanied emotions of shame, will increase the risk of this coping behavior to avoid these experiences. Shame is a highly aversive emotion, which may lead the person to do whatever is necessary to avoid it. Additionally, performance-based self-esteem, which is a risk factor for burnout [98], likely increases the risk for the described coping behavior. It is important to also regard the fact that external factors such as failures leading to highly negative consequences, can increase the likelihood of the described coping behavior.

### 2.4. Burnout Maintaining Behaviors 

The burnout process does not end with clinical burnout (i.e., exhaustion) syndrome: The symptoms, often accompanied by sick-leave, also function as stimuli, often stress-stimuli (i.e., stressors), for different overt and covert behaviors, which potentially maintains the prolonged stress reactions, insufficient recovery processes, and clinical burnout syndrome, particularly among people who on a recurring basis act in accordance with the behavioral patterns described above (e.g., maladaptive perfectionism). Thus, burnout recovery may not take place even if the individual is no longer exposed to the stressors that were responsible for the development of the syndrome.

The clinical burnout syndrome means a general incapacity to cope with demands at home and at work, which will likely lead to stress responses, not only due to the current situation, but also to the unpredictable future situation. Perseverative cognitions may be one of the most important factors maintaining the prolonged/high frequent stress and the lack of recovery in this situation, since there are many things that the person can worry and ruminate about such as past failures and possible future failures, what other people think of the person being burned out, and on sick-leave as well as the prospectively unpredictable health situation. Individuals with low thresholds for anger and hostility may ruminate about many aspects in life, for example, bad conditions at work such as workplace injustice. Additionally, unclear guidance from health care professionals can contribute to perseverative cognitions. For example, the common general advice of only taking it easy and allowing the recovery to take its time may increase worry among performance-oriented individuals. The new context may also lead to general perceptions of stress as well as unsafety, which may contribute to chronic stress reactions [52], and a general reduction in sense of coherence, a factor which has been shown to function as a burnout protective factor [99]. Moreover, although the possible sick-leave situation often means that there is no lack of time to perform necessary tasks, many patients report that they still often experience feelings of time pressure and associated tension, “as if” there was time pressure [4].

Another factor related to stress and well-being is the individual’s financial situation [100]. Being on sick- leave normally entails that one’s income is reduced, sometimes drastically, which most likely leads to stress for a majority of individuals, not only related to the current situation, but also to an uncertain economic future. For example, one of my former clients was often worried about being homeless as a consequence of the predicted inability to pay the rent. Accordingly, a study by Collins et al. [101] showed that workers with paid sick-leave in comparison to workers without paid sick-leave had better sleep quality. Inability to perform and being away from work can also lead to job insecurities. Role as well as relationship conflicts at home can also emerge. For example, should the person staying at home due to burnout and sick-leave do more or less household chores?

Burnout includes cognitive exhaustion [102], often including deficits in some certain memories and executive functions [23]. This not only prevents the person from functioning properly at home and at work, but it can also lead to high levels of worry and catastrophic thoughts, for example, about having Alzheimer’s disease and associated arousal. Accordingly, a study by Jacobsen et al. [103] demonstrated that postintervention maladaptive metacognitive beliefs and postintervention cognitive confidence among individuals on sick-leave due to chronic fatigue were related to postintervention self-reported cognitive impairments, controlling for pre-treatment impairments and pre-treatment meta cognitive beliefs and pre and postscores regarding symptoms. 

Not being able to cope with private life and working life, being affected by psychophysiological ill-health, and being on sick-leave can lead to reduced levels of self-efficacy, increased levels of self-criticism, and accompanied feelings of shame, especially among people living in contexts where high demands are placed on them or among people with high internal demands, which is a common factor between type A behavior, maladaptive perfectionism, and over-commitment to work. Shame involves a focused attention on one’s own emotional pain [104] and if shame is prolonged or highly frequent (several months), it contributes to increased stress responses (for example, measured by cortisol levels) [105]. Shame has been shown to predict poor well-being such as depression (*r* = 0.43) [104]. Shame may also undermine social interactions and associated social support, which is a well-known stress buffering factor [106]. Self-criticism is a contrast to self-compassion, which increases positive affects as well as decreases negative affect and stress responses. Shame is related to both constructive approach coping and maladaptive avoidant coping. The risk of the latter increases if one’s social image or mistakes are difficult to repair [107], which may often be the case for people suffering from clinical burnout. Multiple maladaptive avoidance behaviors may be used in order to cope with self-criticism and shame such as withdrawing from people, minimizing social interactions when meeting people, postponing the return to work, or if returning to work, trying to perform as much as they did before experiencing exhaustion. A covert way to cope with shame is to counteract the self-critical cognitions by disputing these cognitions. However, this avoidant coping behavior potentially leads to rumination [108] and shame in combination with rumination may leave the individual at particular risk of depressive symptoms [109]. Furthermore, social withdrawal behaviors can lead to loneliness, which is associated with exaggerated blood pressure and inflammatory reactivity to acute stress, which is suggested to be a biological mechanism through which loneliness has an impact on health [110]. An additional stress factor among people with clinical burnout is to legitimize their work-related distress and absence from work and restore their morally worthy identities [111]. 

Depression is explained by a nonbeneficial change of context [30]. Exhaustion, being on sick-leave, withdrawal from people, experiences of defectiveness and shame, and so on are contextual factors that may lead to depressive reactions. Depression is accompanied by changes in sympathetic and parasympathetic responses [112,113]: it initially involves sympathetic overactivation (indicating increased stress reactivity) while parasympathetic reduction (indicating decreased stress recovery) begins after a while. It has also been shown that the resting heart rate is higher among depressed than nondepressed people [112], which can interfere with recovery after a period of effort and stress. Furthermore, depression is associated with dysregulation in the HPA-axis (e.g., in the form of increased cortisol levels) [114]. Depressed individuals have also been shown to have a less dynamic and less responsive cortisol activity. Put in other words: cortisol levels in nondepressed individuals increase more in stressful situations and deactivates more at the recovery phase after the stressful period [115]. The fact that neurobiological changes that occur during situations of chronic and mild stress also occur during depression adds to the argument of defining depression not only as an affective behavior, but also a stress condition/behavior [116]; a stress behavior that is probably not likely in general to be of high intensity, but instead prolonged and repeated and likely to undermine recovery processes. Depression is also related to passive behavior such as sedentary behaviors and low levels of exercising, which is problematic since passive behavior run the risk of ill health and low well-being being maintained [30]. Accordingly, an increase in physical activity seems to be beneficial for burnout as well as for depression and anxiety. Effects of physical activity on burnout can hypothetically be explained (mediated) in several ways, for example, via normalization of cortisol levels [117], increased heart rate variability [118], reduction in stressful depressive thinking and increased self-efficacy [119], social contacts and sense of self-worth [120], increased ability to deactivate after stress exposure [88], and improved sleep [121].

A behavior neglected in the context of clinical burnout is phobic behavior, which may be particularly possible to learn in contexts of chronic stress and burnout [4]. Examples of phobic behaviors that can add to the total burden of problems among people with clinical burnout is workplace phobia [4,122] and phobic fear and avoidance of demands and stress reactions [4]. These phobias may highly impair the individual’s ability to function. Related to phobic behavior is more easily triggered stress responses due to classical conditioning [123], which can increase stress reactivity and decrease stress recovery abilities. 

An additional behavior worth noting is hyperventilation, since the majority of patients with clinical burnout syndrome also have hyperventilation syndrome [124]. The severity of the hyperventilation syndrome has been demonstrated to be highly correlated with levels of exhaustion as well as depression, anxiety, sleep disturbances, and quality of life. Prolonged hyperventilation can probably be explained in several ways. One possible explanation is that it fulfills an avoidant function: that of inhibiting or reducing worry (i.e., making the person not feel unprepared to handle an emerging unpredictable stressor).

Not enough sleep is related to exhaustion [81] and the development of clinical burnout [36], long-term sick-leave [125] as well as a lack of recovery from burnout [82]. Whereas some people with clinical burnout do not sleep enough, some sleep excessively (sometimes up to 12 h per night). There is a lack of evidence for this sleep behavior to be adaptive for the recovery of burnout, and there is a wide range of opinions within health care whether such sleep patterns should be supported or intervened. A study by Grossi et al. [126] showed that 14% of the patience referred to treatment for exhaustion disorder/clinical burnout, mostly or always slept ≥9 h every 24 h. They were more often on sick-leave, reported more depression and fatigue, and experienced more daytime sleepiness than those patients who more seldom or never slept ≥9 h. A hypothesis is that excessive sleep for extended periods of time may maintain burnout and accompanied symptoms such as depression [4]. In line with this, Kaplan and Harvey state that hypersomnia is an important mechanism contributing to the maintenance of mood disorders [127]. Moreover, both little and excessive sleep are related to sick-leave and the optimal sleep duration regarding the risk of being absent due to illness is 7.7 h [128].

The most prominent common factor for the behaviors described above are probably their avoidant functions. Avoidance behaviors are associated with not only burnout, but general ill psychological health. The more reduced an individual’s well-being is the more the individual may use avoidance coping, which may deteriorate the well-being. Accordingly, high levels of avoidance behaviors do not only moderate the association between stress and well-being/health, but also predicts more chronic problems [65].

An additional potential behavior problem, both in the development and the maintaining phases, is lack of the awareness of stress responses. People may not be aware of most of their stress-related cognitive processes and unconscious stress is possibly responsible for a considerable part of prolonged stress-related physiological activation [129]. Moderate levels of physiological activation may particularly be unnoticed among people habituated to chronic stress, and during off-work and sick-leave since stress is primarily associated with work. Lack of awareness is especially a problem since “mini stress” does not create alarm signals that motivates for active coping, which is why the stress responses can continue for a long time [130]. The clinically burned-out person may also believe that his/her organism is in recovery, and erroneously conclude that the burnout condition is irreversible because improvements in exhaustion reduction do not occur. This conclusion can lead to more nonbeneficial behaviors such as worry and helplessness. Finally, a problematic situation consisting of exhaustion, sick-leave, and many stressors may lead to health risk behaviors (e.g., unhealthy eating) [83], which can be nonbeneficial for burnout recovery. Accordingly, health behavior improvements accompany burnout reduction [131].

## 3. Discussion

Burnout symptoms among people diagnosed as clinically burned out often last many years [19]. A common assumption is that recovery from burnout is a lengthy process by nature. A complementarily or contrasting perspective is the commonly used perspective in cognitive behavioral therapy, which postulates that the development of a syndrome is often explained differently than the prolongation of the syndrome. This perspective has been neglected in research and literature on burnout. In this paper, based on cognitive and behavioral principles, stress theory, and empirical data regarding stress and burnout, I am suggesting a theoretical model that explains how clinical burnout can be developed and maintained. The model suggests that a reciprocal process between effort, accompanied by an increase in stress responses and decrease in recovery processes, and fatigue may lead to clinical burnout. The primary suggestion of the model is that experiences of severe burnout symptoms and being on sick-leave may lead to continuation of the stress responses and the impaired recovery responses, and consequently the clinical burnout syndrome. The model also suggests that work stress relief may not be enough for clinical burnout recovery to occur. Several behaviors are the same in the development phase as in the maintenance phase such as worry, however, the stressors may be different in the maintenance situation. In addition, new problem behaviors may be frequent in the maintaining phase such as shame, depressive, social withdrawal, work place phobic, hyperventilation, maladaptive metacognitive beliefs, and excessive sleep behaviors. 

In order to accomplish recovery from clinical burnout, maintenance factors of clinical burnout may need to be analyzed and intervened. Both cognitive behavioral therapy aiming at changing several behaviors [11] and solely recovery facilitating behaviors [35] have shown to reduce burnout and comorbid symptoms. Sleep restriction is an effective intervention for insomnia [132]. Regarding hypersomnia, there is unfortunately, to the best of my knowledge, only anecdotal evidence that restricting sleep can lead to positive effects, regarding, for example, energy and function, for people with burnout who sleep excessively [4]. For the change of phobic behaviors, exposure interventions may be motivated [4,66] and behavior activation may be considered for depression [133]. There are different interventions that may work for perseverative cognitions such as excessive worry, for example applied relaxation [134] and exposure [135]. Since problematic behaviors usually covary, intervening one will probably lead to changes in several behaviors [63]. 

In therapeutic situations, the visualization of the proposed theoretical model (see Figure 1) can be used. Patients can receive two models; one in which examples of possible burnout maintaining behaviors (in box 6 in the model) are included, and one without such behaviors included. The latter model gives room for the patient and the professional care provider to formulate behaviors (and interactions between those) that hypothetically maintain the individual’s burnout symptoms. Based on these hypotheses, one can decide how to best attempt to break the maintenance. These hypotheses can then be tested by using single-subject experimental methodology [136]. 

The described model has important implications for society, not only for patients and clinicians in clinical situations, but also for others, for example, scientists, scientific journals, and policymakers such as to (1) facilitate preventive interventions (which is motivated by much other than the proposed model) to avoid the perhaps most problematic stressor (i.e., clinical burnout) the individual has experienced, (2) be careful with long-term full-time sick-leave due to clinical burnout and not jump to the conclusion that prolonged full time sick-leave (rather than changes in context and behavior) is needed when one fails to observe any burnout recovery, (3) offer individuals who are experiencing clinical burnout professional support to assess clinical burnout as a stressor, identify potential burnout maintaining behaviors (as opposed of, for example, relying solely on sick-leave leading to burnout recovery), and accomplish behavioral changes if needed (instead of only giving the common general advice to relax, avoid stress and be self-compassionate), and (4) conduct research on how clinical burnout can be maintained and facilitate such research (e.g., a journal could publish have a special issue focusing on the subject and research funders could particularly fund such research).

In this paper, I have described behaviors that may occur as a consequence of clinical burnout and that have the potential of contributing to the continuation of the long-term stress responses, deficient recovery, and thus perpetuate the burnout syndrome. There is no ambition for the number of behaviors to be exhaustive. In contrast, I hope that the proposed theoretical model that has been described in this paper will encourage researchers to conduct studies in which maintaining factors are analyzed, and, on the basis of the results of these studies, the contents of the model may be revised. 

The model is primarily a theoretical framework that distinguishes between factors contributing to the development and maintenance of the syndrome. However, two variables (the latter in particular) within the framework are critical: (1) overuse of increased effort as a way to cope with a nonbeneficial change regarding stressors/demands and/or resources, and (2) clinical burnout syndrome and accompanied contextual factors (e.g., long-term sick-leave) as a significant stressor potentially leading to sustained stress responses, lack of stress recovery processes, and maintenance of the syndrome. Since the hypothetical importance of these variables for the development and maintenance of clinical burnout have not been directly tested yet, this needs to be done in order to validate (or invalidate) the proposed theoretical model. For example, in a longitudinal study, a way to test the prediction that burnout is a significant stressor potentially leading to self-maintenance is to measure stress reactions in relation to a developed clinical burnout syndrome and how long it takes for a significant level of burnout recovery to occur. High levels of these stress reactions (controlling for variables such as levels of burnout) needs to prospectively predict longer recovery time to support the proposed model. Since the primary aim of the model is pragmatic, to facilitate recovery from clinical burnout, it is of primary importance to test the pragmatic value of the model. This could, for example, be done by comparing an intervention based on a traditional view of burnout where burnout is only considered a consequence of long-term stress and where it is assumed that burnout naturally takes a very long time to recover from, even in the absence of current stressors and stress behaviors, and an intervention based on the proposed CBT-model where burnout is also considered a stressor and where current behaviors risk leading to maintenance of the syndrome. For the proposed model to be supported, the latter intervention must be more effective than the former. 

## 4. Conclusions

In this article, I have described a model that hypothesizes that clinical burnout (and accompanied factors such as long-term sick-leave) is not only a possible consequence of but also a stressor for long-term stress and lack of recovery that potentially leads to maintenance of the syndrome. The model has been motivated, for example, by the fact that clinical burnout is associated with significant symptoms for many years, the lack of evidence that would suggest that clinical burnout can last for many years in the absence of current significant stressors and stress behaviors, and that “the golden standard” of psychological treatments (i.e., CBT) are characterized by using models that describe both how the syndrome is developed and maintained. I have also motivated the model through suggestions of content, particularly in terms of behaviors that are associated with stress responses and that reasonably can arise in the context of clinical burnout such as depression, shame for being clinically burned out, and worry about the worsened financial situation and acquired cognitive deficits. Furthermore, examples of important implications of the model have been described. Finally, the need for testing of the model directly in order to validate it has been emphasized.

## Data Availability

Not applicable.
